# Effects of health on changing labor force participation in Pakistan

**DOI:** 10.1186/2193-1801-2-610

**Published:** 2013-11-15

**Authors:** Ayesha Mushtaq, Asma Mohsin, Khalid Zaman

**Affiliations:** Department of Management Sciences, COMSATS Institute of Information Technology, Abbottabad, Pakistan

**Keywords:** Labor force participation rate, Health expenditures, Secondary school enrolment, Life expectancy at birth, Pakistan

## Abstract

**Abstract:**

The objective of the study investigates the effects of health on changing labor force participation during Pakistan’s economic transition in the 1980s, a period of several economic liberalization and international integration on the health and financial sectors. The study employed the autoregressive distributed lag (ARDL) co-integration technique to estimate the short- and long-run elasticities, while the Wald coefficient restrictions tests was used to determine the dynamic short-run causality between the variables over a period of 1975–2011. The study was limited to a few variables, including age dependency, health expenditures, trade openness, population per bed, life expectancy, gross capital formation, mortality rate, secondary school enrolment and labor force participation rate, in order to manage robust data analysis. The results suggest that infant mortality rate (IMR), gross capital formation (GCF) and secondary school enrolment (SSE) decrease the labor force participation rate in the long-run, as if there is one percent increase IMR, GCF and SSE, labor force participation decreases by 0.653 percent, 0.137 percent and 0.220 percent respectively, however, these results invert the relationship in short-run. The study also finds that health expenditures has positive and significant impact on labor force participation rate in the short-run, but this result disappear in the long-run. Trade liberalization has a positive effect in the short run, while a negative effect is observed in the long run upon labor force participation rate of Pakistan. The study confirms that Pakistan did not enjoy substantial growth benefits related to health care because human capital (secondary school enrolment), trade openness, public investment and infant mortality rate have a negative impact on labor force participation rate. These findings have important policy implications.

**Jel codes:**

H51, I21, J21.

## Introduction

Health is a key factor in a person’s ability to develop his skills and knowledge. The mix of skills, knowledge and capabilities that a person possesses (his human capital) is optimistically related to his productivity and the demand for his labor. Poor health is a hurdle towards developing or using skills, then improving health could raise labor force participation and economic output (Holt, 2010a). In addition, poor health reduces the number of hours worked, or lowers productivity when at work, and then further output could be lost. The costs of treating poor health and the value of lost output are measures of the economic cost of ill health (Holt, 2010b). Therefore, health is one of the most important assets a human being possesses. If these assets erode or are not fully developed, it can cause physical and emotional harm, resulting in obstacles in people's lives. The previous connection can be viewed as the relationship between income and health. Life cycle models have explained how health may determine the future income, wealth and consumption (Lilliard and Weiss, 1997; Smith 1999).

Since its creation Pakistan has exhibited a continuously high rate of population growth. When measured by population size it has moved from the thirteenth largest country in 1950 to the sixth largest country in 2011. According to World Bank projection it will become the fifth largest country by 2050. This rapid increase in population leads to greater demand for food, infrastructure, and services and puts an enormous strain on food security and provision of basic services. Due to rapid population growth and lack of well-developed human resources, Pakistan is faced with socioeconomic crises including food insecurity, and unemployment (GoP 2012). Some of the selected demographic indicators for the period (2010–11 and 2011–12) are posted in Table 1.

**Table 1:** **Selected demographic indicators**

Decades	1980s	1990s	2000s	2012
Population (Million)	96.3	124.6	150.9	180.7
Labour force (Million)	11.6	35.1	45.5	47.1
Employed labour force (Million)	11.2	33.1	42.4	44.3
Unemployed labour force (Million)	0.4	2.0	3.6	3.8
Crude birth rate (Per 1000 Person)	-	-	27.4	27.2
Crude death rate (Per 1000 Person)	-	-	7.9	7.2
Infant mortality rate (Per 1000 Person)	-	-	79.6	74.2

Source: Pakistan Employment Trends (2011) and Government of Pakistan (GoP 2012).

Specifically, the objective of this study is to analyze the impact of health on person’s participation in the labor force. The more specific objectives to estimate whether there is a long-run relationship between health related indicators and labor force participation rate in Pakistan. In addition, to estimate the dynamic short-run causality effects of health indicators towards labor force participation rate in Pakistan. A better understanding of the relationship between health and labor market outcomes is necessary to estimate the costs of health limitations to the economy. This study aim to explore this area as limited work has been done in this field in Pakistan. Moreover, most of the available literature focuses on impact of health on female labor force participation rate. The present study focuses on impact of health on overall labor force participation rate including both males and females in context of Pakistan.

The empirical analysis of health indicators on labor force participation rate has always been of great concern to health economists. Though the area is quite unexplored in Pakistan but there exists enough international literature on the exploring the impact of health indicators on labor force participation rate. Bloom et al. (2009) estimate the effect of fertility on female labor force participation in a panel of countries. The result find that removing legal restrictions on abortion significantly reduces fertility and estimate that, on average, a birth reduces a woman’s labor supply by almost 2 years during her reproductive life. Cai (2010) employs a simultaneous equation model to explore the relationship between health and labour force status in a panel data set of 13,969 Australia (HILDA) household Survey. The result shows that health has a positive and significant effect on labour force participation for both males and females. As for the reverse effect, it is found that labour force participation has a negative effect on male health but a positive effect on female health. Kippersluis et al. (2010) investigate whether the socio economically disadvantaged, on top of a lower health level, experience a sharper deterioration of health over time. Data are taken from the Dutch Central Bureau of Statistics (CBS) Health Interview Surveys covering the period 1983–2000. The result suggests that the widening gradient is attributable both to health-related withdrawal from the labor force, resulting in lower incomes, and the cumulative protective effect of education on health outcomes. The less educated appear to suffer a double health penalty in that they begin adult life with a slightly lower health level, which subsequently declines at a faster rate.

Farid et al. (2012) trace out the human capital related factors which determine employment in Pakistan based on primary source of data, which is collected from 494 households of district Bahawalpur, Pakistan. The study concludes that the completed years of education, experience, various level of education, health status of workers significantly influenced the labor force participation and employment. Robroek et al. (2012) examine the role of poor health, unhealthy behaviors, and unfavorable work characteristics on exit from paid employment due to disability pension, unemployment, and early retirement among older workers. Respondents of the longitudinal Survey of Health, Ageing, and Retirement in Europe (SHARE) in 11 European countries has been selected during the 4-year follow-up period (N=4923). The result shows that poor health was a risk factor for disability pension, and a lack of physical activity was a risk factor for disability pension and unemployment. A lack of job control was a risk factor for disability pension, unemployment, and early retirement. Bayanpourtehrani and Sylwester (2012a) empirically examine associations between female labor force participation and democracy by using a cross-country, time series (1980–2005) data set. The result shows that female labor force participation is lower in democracies, however, the study further finds that the ratio of female labor force participation to male labor force participation is similar under both types of regimes and that male labor force participation is also lower in democracies. Bayanpourtehrani and Sylwester (2012b) further examines whether female labour force participation (FLFP) in a cross-section of countries between 1985 and 2005 varies depending upon the religion practiced in these countries. The result shows that female labour force participation rate is lower in Muslim countries. However, the association between Islam and female labour force participation rate greatly diminishes once other controls are included in the regression, suggesting that Islam might not diminish female labour force participation rate. Moreover, the association between Islam and female labour force participation rate is similar to that between Catholicism and female labour force participation rate. Countries where Protestantism is prevalent or where no religion is practiced have higher female labour force participation rate. Finally, study find some evidence that the association between female labour force participation rate and religion is weakening over time. Table 2 reports some recent studies on between health indicators and labor force participation in the context of Pakistan.

**Table 2:** **Summary of selected studies on health indicators and labor force participation in Pakistan**

Study	Country	Period	Methodology	Results
Hou and Ma (2012)	Pakistan	Pakistan Social and Living Standards Measurement Survey (2008)	Correlation	Women’s decision-making power has a significant positive correlation with maternal health services uptake and that influential males’ decision-making power has the opposite effect.
Chaudhary et al. (2012)	Pakistan	1996-2009	Granger causality test	Consciousness of women about their rights, economic empowerment of women and women’s overall development have positive and significant effect on women’s empowerment.
Farid-ul- Hasnain et al. (2012)	Pakistan	Qualitative study (Questionnaires)	Focus group discussions	Equality among young adults, pointing towards an increasing, sound interaction between the sexes and aspirations for more gender equal relationships.
Khilji et al. (2012)	Pakistan	1980-2010	Cointgration Test	Increase in labour force participation rate found to be helping in increasing the GDP growth rate, vocation training and literacy rate in the country.
Ahmad (2012)	Pakistan	Patient based survey	Multivariate logistic regression model	Incidence of adverse pregnancy outcomes and symptoms of common mental health problems for women during the postnatal period and low weight births and malnutrition among neonates.

In short, it is concluded that poor health whether it is physical health or mental health has negative impact on labor force participation rate, hence there is a pressing need to evaluate and analyze the health-participation nexus and to find out the inter relationship. In the subsequent sections, an effort has been made to empirically find out the short- and long-run relationship between health indicators and labor force participation rate in the context of Pakistan.

The study arrange in the following manners: after introduction which is presented in Section 1 above, Data Source and Methodological Framework are included to share vision with the reader in Section 2. Results and Discussion are in the Section 3. Summary and Conclusion of the study are in the last.

### Data and methodological framework

In order to determine the relationship between health indicators and labor force participation, different health related variables are used in this study. There are three broad categories of health indicators i.e., health input indicators, health output indicators and other related indicators. Health input indicators comprises of health expenditures and availability & quality of health facilities i.e., population per bed doctors. Health output indicators includes life expectancy, infant mortality rate and age dependency ratio while, other health care related expenditures includes trade openness, gross capital formation and school enrolment. The brief description of all the variables used in the study is presented in Table 3. The data of all the variables is used ranging from 1975–2011.

**Table 3:** **Description of variables**

Variables	Measurement	Definition	Expected signs	Data source
Labor force participation rate (LFPR)	Percentage	Normally, the labor force of a country consists of everyone of working age (typically above a certain age (around 14 to 16) and below retirement (around 65) who are participating workers, that is people actively employed or seeking employment. People not counted include students, retired people, stay-at-home parents, people in prisons or similar institutions, people employed in jobs or professions with unreported income, as well as discouraged workers who cannot find work.		GoP (various issues)
**Independent Variables (Health Inputs)**				
Health Expenditures (HEXP)	As percentage of GDP	Total health expenditure is the sum of public and private health expenditure. It covers the provision of health services (preventive and curative), family planning activities, nutrition activities, and emergency aid designated for health but does not include provision of water and sanitation.	Positive	WDI (2012)
Population per bed - Doctors	Physicians per 1000 people	Hospital beds comprise those available in public and private, general and specialized hospitals, and rehabilitation centers. Hospitals are establishments permanently staffed by at least one physician.	Positive	WDI (2012)
**Health Output Variables**				
Age dependency ratio (ADEP)	Percentage	The dependency ratio is an age-population ratio of those typically not in the labor force (the dependent part) and those typically in the labor force (the productive part). It is used to measure the pressure on productive population.	Negative	WDI (2012)
Life Expectancy at birth (LE)	Years	Life expectancy at birth indicates the number of years a newborn infant would live if prevailing patterns of mortality at the time of its birth were to stay the same throughout its life.	Positive	WDI (2012)
Infant Mortality Rate (IMR)	Per 1000 individuals per year	Mortality rate is a measure of the number of deaths in a population, scaled to the size of that population, per unit of time. Mortality rate is typically expressed in units of deaths per 1000 individuals per year.	Negative	WDI (2012)
**Others Health Related Indicators**				
Trade Openness (TOP)	Exports plus imports as percentage of GDP	Trade openness is the removal or reduction of restrictions or barriers on the free exchange of goods between nations. This includes the removal or reduction of both tariff (duties and surcharges) and non-tariff obstacles (like licensing rules, quotas and other requirements). The easing or eradication of these restrictions is often referred to as promoting “free trade.”	Positive	WDI (2012)
Gross Capital Formation (GCF)	As percentage of GDP	Gross capital formation (formerly gross domestic investment) consists of outlays on additions to the fixed assets of the economy plus net changes in the level of inventories.	Positive	WDI (2012)
Secondary School Enrolment (PSE)	Percentage of Gross enrolment	Secondary school enrollment is the proportion of the population of the official age for secondary education according to national regulations who are actually enrolled in secondary schools.	Positive	WDI (2012)

Source: FBD (various issues) and WDI (2012).

**Figure 1:**
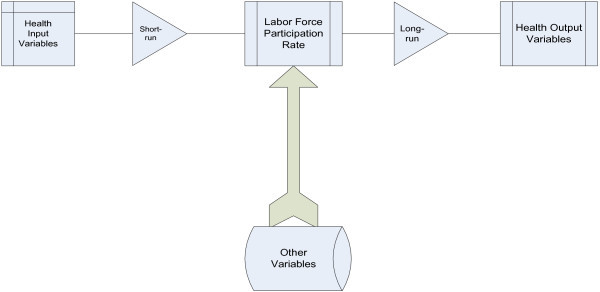
**Research framework.**

Figure 1 shows the conceptual framework for health indicators and labor force participation rate in Pakistan. These connections show that health variables have short and long-run connection with labor force participation rate in Pakistan. Government should have to allocate health care budget for healthier and prosperous economy.

#### Theoretical model

Given the importance of labor in endogenous growth theory and the fact that health determines the quality of labor supply; the causal relationship between labor force participation and health have crucial role to play in determining the productivity of labor force for the long term requirements of economic growth. The poor health and low participation rate may have adverse effect on the performance of an economy. The reasons could be in two folds: unhealthy potential work force may impose a cost in terms of production loss by restraining its population at large from participating in the labor force or through reduced labor productivity. Secondly, there could be loss of revenue in terms of cost incurred in providing health care services to maintain good health without any incentive (Pandey, 2009).

Numerous models have been developed to incorporate impact of human capital in economic growth. Romer (1990) and Barro (1991) have emphasized that health is the most important factor in determining the labor force participation. As the focus of study is to analyze the effects of human capital on labor force participation, therefore, the human capital is separated into two parts i.e., health human capital (H) and other forms of human capital i.e. education human capital (E). Labor force participation (Y) is assumed as a function of the stocks of physical capital (K), health human capital (H), education human capital (E) and a vector of other variables (Z) that include technology and other environmental variables.1

Where Y is labor force participation, H is health human capital, E is Education human capital and Z represents all other explanatory variables. *H* in time *t* is the sum of the stock of health human capital in the previous period and accumulation to the stock in the current period. It is assumed that accumulation in the health human capital stock (H) depends on the amount of resources devoted to health care and the efficiency by which this expenditure is converted into health stock. It is further assumed that quantity of resources devoted to health investment is a product of the proportion of income devoted to health care (*YH*) and the level of income. The stock of health human capital evolves in the following way.2

Where λ is the productivity parameter of health expenditure and all other variables. The ability to transform health expenditure into health stock is assumed to be dependent on the stock of health human capital. The health technology equation can be written as:λ=λ (H). Substituting λ into the ∆H equation and that in turn into the production function, the income growth equation become.3

### Econometric model

#### Autoregressive Distributed Lag Model (ARDL)

The traditional approach to determining long run and short run relationships among variables has been to use the standard Johnson Cointegration and VECM framework, but this approach suffers from serious flaws as discussed by Pesaran et al. (2001). Johnson cointegration test will not give you a consistent way of deciding the cointegration rank. As independent variable is I(1) while dependent variables are I(0) and I(1) as explained previously so most appropriate technique is ARDL(Autoregressive Distributed Lag Model). As this type of dynamic model is part of the Koyck distribution class of models. It is used in models where adjustment does not occur immediately, but takes a number of time periods to fully adjust. W e can apply a specific restriction to a general ARDL model to determine if partial adjustment is taking place. This model has as its dependent variable a desired value or planned value. This desired value is then determined by the usual explanatory variables.

In order to obtain results, we utilize the ARDL approach to establish the existence of long-run and short-run relationships. ARDL is extremely useful because it allows us to describe the existence of an equilibrium/relationship in terms of long-run and short-run dynamics without losing long-run information.

Following Pesaran et al. (2001), we assemble the vector autoregression (VAR) of order *p,* denoted VAR (*p*), for the following growth function:4

where *z*_*t*_ is the vector of both *x*_*t*_ and *y*_*t*_ , where *y*_*t*_ is the dependent variable defined as labor force participation rate (LFPR), *x*_*t*_ is the vector matrix which represents a set of explanatory variables and *t* is a time or trend variable. According to Pesaran et al. (2001), *y*_*t*_ must be I(1) variable, but the regressor *x*_*t*_ can be either I(0) or I(1). We further developed a vector error correction model (VECM) as follows:5

where ∆ is the first-difference operator. The long-run multiplier matrix λ as:

The diagonal elements of the matrix are unrestricted, so the selected series can be either I(0) or I(1). If λYY=0, then *Y* is I(1). In contrast, if λYY<0, then *Y* is I(0).

The VECM procedures described above are imperative in the testing of at most one cointegrating vector between dependent variable *y*_*t*_ and a set of regressors *x*_*t*_. To derive model, we followed the postulations made by Pesaran et al. (2001) in Case III, that is, unrestricted intercepts and no trends. After imposing the restrictions λ_YY_=0, μ≠0 and *α=*0, the hypothetical function can be stated as the following unrestricted error correction model (UECM):6

Where ∆ is the first-difference operator and *u*_*t*_ is a white-noise disturbance term. Equation (6) also can be viewed as an ARDL of order (p, *q*, *r, s, t, u, v, w, x*). Equation (6) indicates that agriculture expenditure tends to be influenced and explained by its past values. The structural lags are established by using minimum Akaike’s information criteria (AIC). From the estimation of UECMs, the long-run elasticities are the coefficient of one lagged explanatory variable (multiplied by a negative sign) divided by the coefficient of one lagged dependent variable (Bardsen, 1989). For example, in Equation (6), the long-run inequality, investment and growth elasticities are (*β*_2_/*β*_1_), (*β*_3_/*β*_1_), *β*_4_/*β*_1_ etc. The short-run effects are captured by the coefficients of the first-differenced variables in Equation (6).

After regression of Equation (6), the Wald test (*F*-statistic) was computed to differentiate the long-run relationship between the concerned variables. The Wald test can be carry out by imposing restrictions on the estimated long-run coefficients of economic growth, inequality, investment and public expenditure. The null and alternative hypotheses are as follows:

*H*_0_ : *β*_1_ = *β*_2_ = *β*_3_ = *β*_4_ = *β*_5_ + *β*_6_ + *β*_7_ = *β*_8_ = *β*_9_ = 0 (no long-run relationship)

Against the alternative hypothesis

*H*_0_ : *β*_1_ ≠ *β*_2_ ≠ *β*_3_ ≠ *β*_4_ ≠ *β*_5_ ≠ *β*_6_ ≠ *β*_7_ ≠ *β*_8_ ≠ *β*_9_ ≠ 0 (long-run relationship exists)

The computed *F*-statistic value will be evaluated with the critical values tabulated in Table CI (iii) of Pesaran et al. (2001). According to these authors, the lower bound critical values assumed that the explanatory variables *x*_*t*_ are integrated of order zero, or I(0), while the upper bound critical values assumed that *x*_*t*_ are integrated of order one, or I(1). Therefore, if the computed *F*-statistic is smaller than the lower bound value, then the null hypothesis is not rejected and we conclude that there is no long-run relationship between poverty and its determinants. Conversely, if the computed *F*-statistic is greater than the upper bound value, then agriculture expenditure and its determinants share a long-run level relationship. On the other hand, if the computed *F*-statistic falls between the lower and upper bound values, then the results are inconclusive.

## Results and discussion

The standard Augmented Dickey-Fuller (ADF) unit root test was exercised to check the order of integration of these variables. The results obtained are reported in Table 4. Based on the ADF test statistic, it was initiate that out of nine variables, five have unit root i.e., LFPR, TOP, GCF, PPBED and HEXPM while other four variables are I(0) i.e., LE, AD, SSE and IFM. Noticeably, the mixture of both I(0) and I(1) variables would not be possible under the Johansen procedure. This gives a good justification for using the bounds test approach, or ARDL model, which was proposed by Pesaran et al. (2001).

**Table 4:** **Results of ADF Test**

Variable name	LEVEL			1^st^DIFFERECE			
	Intercept	Trend	None	Intercept	Trend	None	Decision
LFPR	−2.285 (−2.642)	−1.975 (−3.254)	−0.322 (−1.608)	−5.493 (−2.646)	−5.915 (−3.261)	−5.588 (−1.607)	Non Stationary at level but stationary at first difference i.e.,I(1)
LE	−0.782 (−2.642)	−4.817 (−3.254)	5.356 (−1.608)	−6.259 (−2.646)	−6.143 (−3.261)	−0.539 (−1.607)	Stationary at level i.e., I(0)
AD	4.504 (−2.642)	2.147(−3.254)	−1.132 (−1.608)	0.407 (−2.646)	−3.817 (−3.261)	0.979 (−1.607)	Stationary at level i.e., I(0)
TOP	−1.817 (−2.642)	−1.892 (−3.254)	−0.697 (−1.608)	−5.171 (−2.646)	−4.869 (−3.261)	−5.259 (−1.607)	Non Stationary at level but stationary at first difference i.e., I(1)
GCF	−2.283 (−2.642)	−2.461 (−3.254)	−0.185 (−1.608)	−4.579 (−2.646)	−4.458 (−3.261)	−4.699 (−1.607)	Non Stationary at level but stationary at first difference i.e., I(1)
SSE	−0.825 (−2.642)	−1.464 (−3.254)	2.374 (−1.608)	−3.896 (−2.646)	0.5140 (−3.261)	−3.029 (−1.607)	Stationary at level i.e., I(0)
PPBED	−1.118 (−2.642)	−0.899 (−3.254)	−1.509 (−1.608)	−4.516 (−2.646)	−4.730 (−3.261)	−4.094 (−1.607)	Non Stationary at level but stationary at first difference i.e., I(1)
IMR	0.3490 (−2.642)	−3.435 (−3.254)	−9.031 (−1.608)	−4.536 (−2.646)	−4.289 (−3.261)	−0.606 (−1.607)	Stationary at level i.e., I(0)
HEXP	−1.465 (−2.642)	−1.724 (−3.254)	−0.403 (−1.608)	−3.575 (−2.646)	−3.660 (−3.261)	−3.636 (−1.607)	Non Stationary at level but stationary at first difference i.e., I(1)

*Note:* The null hypothesis is that the series is non-stationary, or contains a unit root. The rejection of the null hypothesis is based on MacKinnon (1996) critical values. The lag length are selected based on SIC criteria, this ranges from lag zero to lag two. Bracket shows the critical t-values.

The ARDL approach estimates (p + 1) ^k^ number of regressions in order to obtain optimal lag length for each variable in model, where *p* is the maximum number of lags to be used and *k* is the number of variables in the regression. The structural lags are selected on the basis of minimum value of AIC (Akaike Information Criteria) and it is shows in Table 5.

**Table 5:** **VAR Lag order selection criteria**

Lag	LogL	LR	FPE	AIC	SC	HQ
0	588.0169	NA	8.83e-30	−41.35835	-40.93014*	−41.22745
1	671.8937	107.8415	9.67e-30	−48.10878*	−37.28175	−40.25476
2	844.5229	110.9759*	1.48e-31*	−41.56383	−39.97281	-45.62153*

* indicates lag order selected by the criterion.

LR: sequential modified LR test statistic (each test at 5% level), FPE: Final prediction error.

AIC: Akaike information criterion, SC: Schwarz information criterion, HQ: Hannan-Quinn information criterion.

The estimation of Equation (6) using the ARDL model is reported in Table 6. Using Hendry’s general-to-specific method, the goodness of fit of the specification, that is, *R-*squared and adjusted *R*-squared, is 0.940 and 0.871 respectively. The robustness of the model has been definite by several diagnostic tests such as Breusch- Godfrey serial correlation LM test, ARCH test, Jacque-Bera normality test and Ramsey RESET specification test. All the tests disclosed that the model has the aspiration econometric properties, it has a correct functional form and the model’s residuals are serially uncorrelated, normally distributed and homoskedastic. Therefore, the outcomes reported are serially uncorrelated, normally distributed and homoskedastic. Hence, the results reported are valid for reliable interpretation.

**Table 6:** **Results of ARDL equation**

Dependent variable: ∆ log (LFPR)_***t***_				
Variable	Coefficient	Std. Error	t-Statistic	Prob.
C	6.336	2.624	2.414	0.094
LOG(LFPR_*t*-1_)	−1.258	0.217	−5.774	0.010
LOG(AD_*t*-1_)	−0.082	0.253	−0.327	0.764
LOG(HEXP_*t*-1_)	−0.012	0.016	−0.722	0.522
LOG(IMR_*t*-1_)	−0.653	0.206	−3.159	0.050
LOG(GCF_*t*-1_)	−0.137	0.033	−4.044	0.027
LOG(LE_*t*-1_)	0.745	0.427	1.745	0.179
LOG(PPBED_*t*-1_)	0.027	0.114	0.241	0.824
LOG(SSE_*t*-1_)	−0.220	0.059	−3.733	0.033
LOG(TOP_*t*-1_)	−0.170	0.032	−5.217	0.013
∆LOG(LFPR_*t*-1_)	0.124	0.084	2.852	0.074
∆LOG(AD_*t*-1_)	−0.019	0.017	−1.088	0.356
∆LOG(HEXP_*t*-1_)	1.017	0.202	5.029	0.015
∆LOG(IMR_*t*-1_)	−0.147	0.026	−5.563	0.011
∆LOG(GCF_*t*-1_)	−0.001	0.233	−2.189	0.074
∆LOG(LE_*t*-1_)	0.179	0.079	2.264	0.108
∆LOG(PPBED_*t*-1_)	0.171	0.044	3.858	0.030
∆LOG(SSE_*t*-1_)	−0.095	0.022	−4.275	0.023
∆LOG(TOP_*t*-1_)	0.442	0.235	2.880	0.032
**11. Model criteria/Goodness of Fit:**				
R-square = 0.940; Adjusted R-square = 0.871; Wald F-statistic = 11.236 [0.001]**				
**111. Diagnostic Checking:**				
JB = 8.172 [0.1334]; LM-1 = 1.064 [0.3117]; LM-2 = 0.705 [0.503]; LM-3 = 0.491 [0.691]; ARCH (1) = 0.238 [0.627]; ARCH-2 = 1.044 [0.360]; ARCH-3 = 0.699 [0.562]; White Heteroscedasticity = 0.404 [0.971]; Ramsey RESET = 2.009 [0.155]				

*Note:* Probability values are quoted in square brackets. MA and ARCH denote LM-type Breusch-Godfrey Serial Correlation LM and ARCH test, respectively, to test for the presence of serial correlation and ARCH effect. JB and RESET stand for Jarque-Bera Normality Test and Ramsey Regression Specification Error Test, respectively.

The results suggest that infant mortality rate, gross capital formation and secondary school enrolment decrease the labor force participation rate in the long-run, however, these results invert the relationship in short-run. The study also finds that health expenditures has positive and significant impact on labor force participation rate in the short-run, but this result disappear in the long-run. Trade liberalization has a positive effect in the short run, while a negative effect is observed in the long run upon labor force participation rate of Pakistan.

In Table 7, the results of the bounds co-integration test demonstrate that the null hypothesis of against its alternative is easily rejected at the 1% significance level. The computed *F*-statistic of 11.236 is greater than the upper critical bound value of 5.06, thus indicating the existence of a steady-state long-run relationship among health indicators and labor force participation rate.

**Table 7:** **Bounds test for cointegration analysis**

Critical value	Lower bound value	Upper bound value
1%	3.74	5.06
5%	2.86	4.01
10%	2.45	3.52

*Note:* Computed F-statistic: 11.236 (Significant at 0.01 marginal values)^a^ *.*

The estimated coefficients of the long-run relationship between health expenditures and labor force participation rate are expected to be significant, that is:

Table 8 indicates that age dependency, health expenditures, infant mortality rate, gross capital formation and secondary school enrolment are negatively correlated to the labor force participation rate, with the estimated elasticities of 0.065%, 0.009%, 0.510%, 0.709% and 0.175% respectively. However, remaining variables are positively associated with the labor force participation rate in Pakistan.

**Table 8:** **Long-run elasticities and short-run causalities between health indicators and labor force participation rate in Pakistan**

**1. Long-run estimated coefficient**					
	**Variable**			**Coefficient**	
	LOG(AD)			-0.065**	
	LOG(HEXP)			-0.009***	
	LOG(IMR)			-0.510**	
	LOG(GCF)			-0.709**	
	LOG(LE)			0.592***	
	LOG(PPBED)			0.022***	
	LOG(SSE)			-0.175**	
	LOG(TOP)			0.135**	
**11. Short-run Causality Test (Wald Test F-statistic):**					
∆*AD*	∆*HEXP*	∆*IMR*	∆*GCF*	∆*LE*	∆*PPBED*
0.595	20.215*	0.516	8.516*	0.516	15.516*
(0.182)	(0.000)	(0.426)	(0.001)	(0.826)	(0.000)
∆*SSE*	∆*TOP*				
11.516*	0.425				
(0.000)	(0.912)				

*, ** and *** denote significant at 1%, 5% and 10% level. Figures in brackets refer to marginal significance values.

The dynamic short-run causality among the relevant variables is shown in Table 8, Panel 11. The causality effect can be acquired by restricting the coefficient of the variables with its lags equal to zero (using Wald test). If the null hypothesis of no causality is rejected, then we wrap up that a relevant variable Granger-caused economic growth.

From this test, we initiate that health expenditures, gross capital formation, population per bed and secondary school enrolment are statistically significant to Granger-caused labor force participation rate in Pakistan at 1% significance level. To sum up the findings of the short-run causality test, we conclude that causality running from health indicators to labor force participation rate.

## Conclusion and policy implications

The main objective of this study is to analyze the impact of various aspects of health on labor force participation rate in Pakistan. To explore impact of health indicators on labor force participation rate, the present study used bounds testing approach to cointegration. The results suggest that infant mortality rate, gross capital formation and secondary school enrolment reduce the labor force participation rate in the long-run, however, these results overturn the relationship in short-run. The study also finds that health expenditures has encouraging and significant impact on labor force participation rate in the short-run, but this result evaporate in the long-run. Trade liberalization has a positive effect in the short run, while a negative effect is experiential in the long run upon labor force participation rate of Pakistan.

The following are some policy suggestions derived from the findings of the study i.e.,

- Health is often regarded as an important factor in individuals’ labor supply decision, not only because health is a form of human capital, valued by both employers and employees (Becker, 1964; Grossman, 1972), but also because individuals’ preferences between work and leisure may change following a health shock. Economists have increasingly recognized that good health across the whole population significantly contributes to labor and human capital to achieve economic growth. Through higher participation and productivity, good health contributes to economic performance and is positive for individual wellbeing (Hsiao and Heller, 2007).
- Dependency burden is a big obstacle in the way of growth and development. Dependency burden may be cut down by providing old age benefits to aged worker and free education and health facilities to children. Population per bed must be decreased it means that number of beds available to the people must be increased so that health facilities should be provided to all the people in a proper manner.
- Investment as a % of GDP must be made in developmental purposes instead of non developmental works. So when investment is made in developmental works it results in employment generation. Thus labor force participation rate can be increased. Access to good health can contribute positively to the economic and social development of a country. Thus, key issues that impact the health status of people ought to be addressed through a diverse set of policy tools comprising short and long term measures to secure better health outcomes (GoP 2012).
- Insufficient health spending and rapid population growth in Pakistan have contributed to continuing low facilities to population ratios particularly in the case of dentists, nurses and hospital beds. The potential pay off of investing in and improving the overall health services is enormous (GoP 2011).
- Health expenditures must be increased and they must be properly utilized means policy direction must be changed in such a way that more people will able to get health care services. When expenditures are properly made than life expectancy at birth will increase which results in increase in labor force participation rate as people have now more time to spend in labor market (Rechel et al. 2009).
- Balanced growth in population is crucial for the welfare of the country or improving the productive capacity of the economy. It is important to know the size of a country’s population, its growth rate and other demographic attributes in order to analyze the dynamics of the population, labour force and employment and to estimate the quantity of goods and services that will be needed to meet future demand (Bloom et al. 2011).

To sum up, overall, health conditions are deteriorating in Pakistan. Only small amount of budget is allocated for this sector i.e. 0.5% of GNP. Due to poor health facilities major portion of the population is facing different disease but they have no proper access to cure them. Due to the poor health most of the people remain out of the labor force. Pakistan’s labor market is showing its inability to continue the past trend of labor absorption (Labour Force Survey 2010–11). The labor market is presently confronted with the twin menace of unemployment and underemployment. The situation in the labor market is serious on yet another account. The working conditions of those lucky found employed, by and large, are not satisfactory, rather they are deplorable. Long working hours and poor working conditions are the normal features of a significant number of work places. A number of them also carry occupational safety and health hazards.

## Endnotes

^a^Critical Values are cited from Pesaran et al. (2001), Table CI (iii), Case 111: Unrestricted intercept and no trend.
